# Ultrastructural Analysis of *Pseudanthus* (Picrodendraceae) Pollen Using Transmission Electron Microscopy: Intraspecific, Interspecific, Intrafamilial, and Interfamilial Comparisons

**DOI:** 10.3390/plants15010061

**Published:** 2025-12-24

**Authors:** Angelika Till, Silvia Ulrich, David J. Cantrill, Friðgeir Grímsson

**Affiliations:** 1 Division of Structural and Functional Botany, Department of Botany and Biodiversity Research, University of Vienna, 1030 Vienna, Austria; silvia.ulrich@univie.ac.at; 2 Royal Botanic Gardens Victoria, Birdwood Avenue, Melbourne, VIC 3004, Australia; david.cantrill@rbg.vic.gov.au

**Keywords:** Australia, endexine, foot layer, infratectum, Malpighiales, pantoporate, *Pseudanthus*

## Abstract

Until now investigations on the ultrastructural characteristics of *Pseudanthus* pollen using transmission electron microscopy (TEM) were limited. The aims of this study were as follows: (1) to present the first comprehensive TEM-based analysis of *Pseudanthus* pollen; (2) to provide a holistic morphological and ultrastructural description of *Pseudanthus* pollen; (3) to compare *Pseudanthus* pollen to that from other Picrodendraceae and closely related families; (4) to clarify intraspecific, interspecific, intrafamilial, and interfamilial character traits of *Pseudanthus* pollen in relation to that from related genera/families; and (5) to conclude if *Pseudanthus* pollen could potentially be identified in the palynological record. *Pseudanthus* pollen samples were collected from anthetic flowers and prepared according to standard methods for investigation with TEM. Interpretations of pollen ultrastructure in other Picrodendraceae and closely related families were based on previously published TEM micrographs. The pollen ultrastructure from six out of nine *Pseudanthus* species is described here for the first time. By integrating LM, SEM, and TEM techniques, this study offers a holistic perspective on the genus’s pollen morphology and ultrastructural range. It also illuminates the intraspecific and interspecific pollen morphological and ultrastructural diversity within *Pseudanthus* and how it differentiates from other Picrodendraceae as well as the Euphorbiaceae and Phyllanthaceae. The combined morphological and ultrastructural traits of *Pseudanthus* pollen render it unique among Picrodendraceae and differentiate it from the pollen of closely related families. This opens the door for future paleopalynological investigations, but until now *Pseudanthus* pollen has not been reported from the fossil record.

## 1. Introduction

*Pseudanthus* Sieber ex Spreng. comprises nine species from the Picrodendraceae Small, a group of monoecious shrubs and subshrubs endemic to Australia [1,2]. The plants thrive in habitats ranging from open eucalypt forests and mallee woodlands to coastal dunes and rocky mountain slopes [3]. Although light microscopy (LM) and scanning electron microscopy (SEM) studies on *Pseudanthus* pollen have been conducted [4,5,6,7], transmission electron microscopy (TEM) investigations on the ultrastructural characteristics of pollen from this genus are mostly lacking. Earlier LM studies on pollen of *P. ovalifolius* F.Muell. and *P. pimeleoides* Sieber ex Spreng by Erdtman [4], Punt [5], and Köhler [8] provided fundamental insights into the basic pollen morphology of the genus. These investigations laid the foundation for understanding the general pollen characteristics within the genus and were followed by Levin and Simpson [6] and Simpson and Levin [9], who applied both LM and SEM to investigate pollen of *P. divaricatissimus* (Müll.Arg.) Benth. This culminated with the combined LM and SEM study by Till et al. [7] comprising the pollen morphology of eight out of the nine currently recognized *Pseudanthus* species. The study by Till et al. [7] showed that the distinctive combination of pollen characteristics in *Pseudanthus*, including pollen size, spherical shape, thickness and interrelation of pollen wall layers, echinate-perforate sculpturing, size and arrangement of sculpture elements, and aperture configuration, is characteristic for Picrodendraceae. Furthermore, the unique combination of LM- and SEM-based morphological features observed for *Pseudanthus* pollen are only partly shared by pollen from three other closely related genera, *Kairothamnus* Airy Shaw, *Scagea* McPherson, and *Stachystemon* Planch. Till et al. [7] also showed that the combined morphological features of *Pseudanthus* pollen can be used to differentiate species and/or species groups within the genus. These authors also suggested that further ultrastructural investigations are necessary to conclude if TEM analysis could add further diagnostic features to pollen of this genus, and aid in the segregation of species and/or species groups. Until now, ultrastructural research on *Pseudanthus* pollen were limited to that from a single species, *P. divaricatissimus*, portrayed by Levin and Simpson [6]. In palynology, advanced imaging techniques such as TEM play a crucial role in visualizing the internal structure of pollen walls, which can be critical for accurate taxonomic identification as it provides important information on the pollen wall ultrastructure that helps distinguish not only between families and/or genera but also species [10]. Therefore, information on the structure of the pollen wall, the interrelation of the wall layers, and potential thickenings in aperture areas, as well as other diagnostic features of *Pseudanthus* pollen, are needed.

Here, the gap in knowledge of the pollen wall ultrastructure within *Pseudanthus* is filled with a comprehensive TEM investigation on the pollen walls from eight out of the nine currently accepted *Pseudanthus* species (Table 1). Complete sections through the pollen wall of each species are provided, along with detailed sections from both aperture and interapertural areas. The structural variation and detailed features of the pollen walls at interapertural areas and the apertures are summarized and compared. TEM-based pollen descriptions are provided for each species and an updated pollen description for the whole genus, comprising also previous LM- and SEM-based analyses, is presented. Additionally, the pollen morphology and ultrastructure of *Pseudanthus* pollen is compared to that of pollen from other Picrodendraceae, as well as to pollen from the closely related families, Phyllanthaceae Martinov and Euphorbiaceae Juss. Finally, the usefulness of *Pseudanthus* pollen in the fossil record is re-evaluated based on the combined morphological and ultrastructural features observed with LM, SEM, and TEM.

## 2. Results

**Figure 1:**
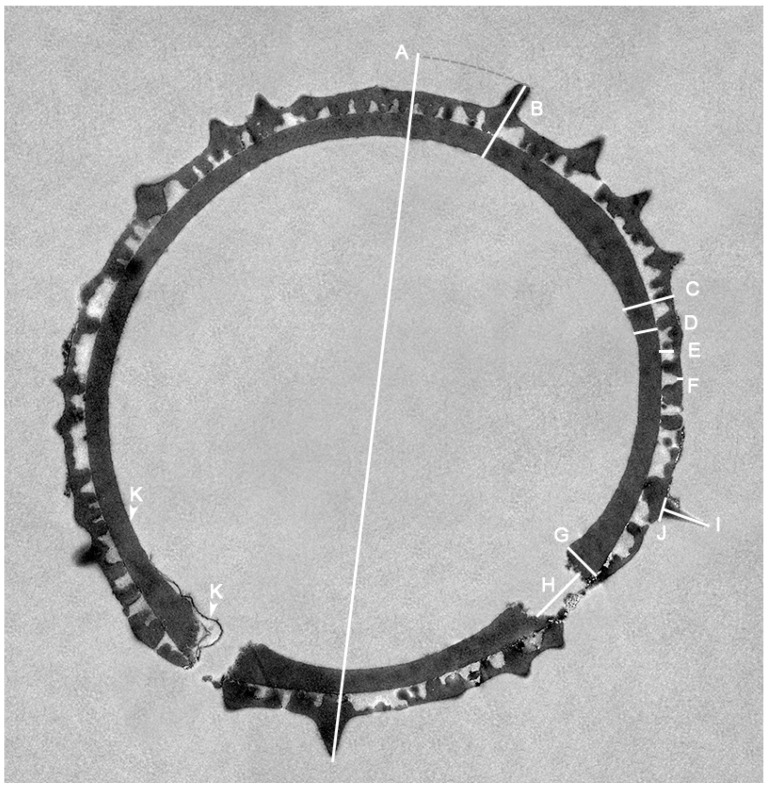
Measuring of *Pseudanthus* pollen from TEM micrographs using Photoshop (25.1.0). **A**. Pollen grain diameter. **B**. Ektexine incl. echini. **C**. Ektexine excl. echini. **D**. Foot layer. **E**. Infratectum. **F**. Tectum. **G**. Foot layer at aperture. **H**. Aperture diameter. **I**. Echinus height. **J**. Echinus width at base. **K**. Endexine, as a thin band in interapertural area and also detached from the aperture due to acetolysis treatment.

Classification at the family level follows the system proposed by the Angiosperm Phylogeny Group [11]. Family- and genus-level taxonomy adhere to Halford and Henderson [3], with *Pseudanthus* now placed within the Picrodendraceae [12]. The pollen descriptions are arranged alphabetically according to the species names. The pollen ultrastructure of each species is described separately, including all main features and measurements. The TEM-based pollen terminology follows Halbritter et al. [13]. Each species description is accompanied by three TEM micrographs showing the following: (1) cross-section through a complete pollen (see Figure 1 as example), (2) close-up of the interapertural area, and (3) close-up of the aperture. The main features and all measurements are also provided in Table 2. The species-based pollen descriptions are followed by an updated whole-genus pollen description combining previous LM and SEM morphological features from Till et al. [7] with those from this TEM study.

### 2.1. Pollen Descriptions

Clades: Eudicots/Superrosids/Rosids/Fabids.

Order: Malphigiales Juss. ex. Bercht. & J.Presl.

Family: Picrodendraceae Small.

Genus: *Pseudanthus* Sieber ex Spreng.

**Figure 2:**
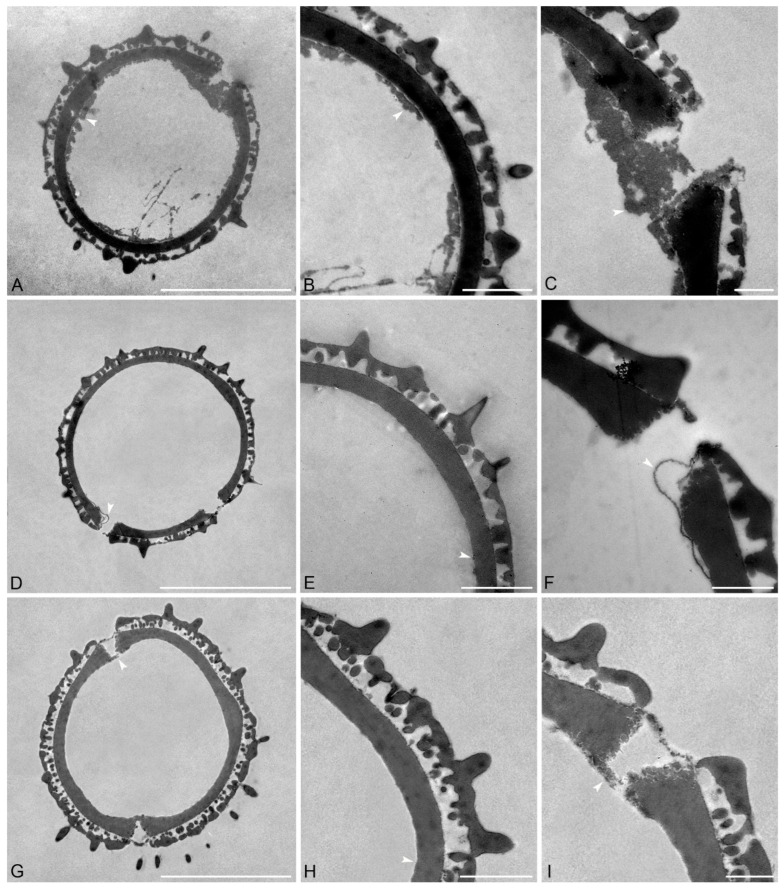
TEM micrographs of *Pseudanthus* pollen. (**A**–**C**) *Pseudanthus divaricatissimus* (MEL2291167). (**A**) Overview, whole cross-section. (**B**) Interapertural area. (**C**) Apertural area. (**D**–**F**) *Pseudanthus ligulatus* subsp. *volcanicus* (MEL1526549). (**D**) Overview, whole cross-section. (**E**) Interapertural area. (**F**) Apertural area. (**G**–**I**) *Pseudanthus micranthus* (MEL625058). (**G**) Overview, whole cross-section. (**H**) Interapertural area. (**I**) Apertural area. White arrows indicate the endexine. Scale bars—10 µm (**A**,**D**,**G**), 2 µm (**B**,**E**,**H**), and 1 µm (**C**,**F**,**I**).

Species: *Pseudanthus divaricatissimus* (Müll.Arg.) Benth. (Figure 2A–C and Table 2)

Description: Pollen diameter incl. echini 18.8–19.9 µm, ektexine incl. echini 2.3–3.7 µm thick, ektexine excl. echini 1.5–2.1 µm thick; ektexine composed of a columellate-tectate pollen wall with supratectal echini, echini 1.3–1.9 µm in height and 0.5–0.7 µm wide at base; eutectum perforate, tectum 0.2–0.4 µm thick; columellate infratectum 0.4–0.5 µm thick; compact-continuous foot layer, 0.8–1.1 µm thick at interapertural areas, foot layer thickened around apertures, up to 1.6–2 µm thick, with frayed granular ends; endexine discontinuous, spongy, 0–1.2 µm tick, also observed in apertural regions, closing the apertures.

Remarks: The pollen of this species has the tallest echini compared to pollen from all other investigated *Pseudanthus* species. Still, the angle of the cut can affect the measurements.

Species: *Pseudanthus ligulatus* subsp. *volcanicus* Halford & R.J.F.Hend. (Figure 2D–F and Table 2).

Description: Pollen diameter incl. echini 16.2–16.8 µm, ektexine incl. echini 2.1–2.2 µm thick, ektexine excl. echini 1–1.2 µm thick; ektexine composed of a columellate-tectate pollen wall with supratectal echini, echini 0.6–0.9 µm in height and 0.7–0.9 wide at base; eutectum perforate, tectum 0.1–0.2 µm thick; columellate infratectum, 0.3–0.4 µm thick; compact-continuous foot layer, 0.6 µm thick at interapertural areas, foot layer thickened around apertures, up to 1–1.1 µm thick, with frayed, lamellar to granular ends; very thin endexine present under the foot layer, 0–0.05 µm thick.

Remarks: The *P. ligulatus* subsp. *volcanicus* pollen is smaller and has thinner ektexine incl. and excl. echini compared to pollen from *P*. *divaricatissimus*. The pollen of *P. ligulatus* subsp. *volcanicus* has the thinnest infratectum (0.3–0.4 µm thick) compared to pollen from all other *Pseudanthus* species.

Species: *Pseudanthus micranthus* Benth. (Figure 2G–I and Table 2).

Description: Pollen diameter incl. echini 20.3–21.1 µm, ektexine incl. echini 2.9–3.9 µm thick, ektexine excl. echini 1.9–2.3 µm thick; ektexine composed of a columellate-tectate pollen wall with supratectal echini, echini 0.9–1 µm in height and 0.7–0.8 µm wide at base; eutectum perforate, tectum 0.3–0.4 µm thick; columellate infratectum, 0.6–0.9 µm thick; compact-continuous foot layer, 0.8–0.9 µm thick at interapertural areas, foot layer thickened around apertures, up to 1.3–1.4 µm thick, with frayed, lamellar to granular ends; very thin and discontinuous endexine layer (0–0.05 µm) present under foot layer, also in apertural regions.

Remarks: The pollen of *P. micranthus* has both the thickest ektexine incl. and excl. echini (up to 3.9 µm and 2.3 µm thick, respectively) compared to pollen from all the other *Pseudanthus* species investigated herein.

**Figure 3:**
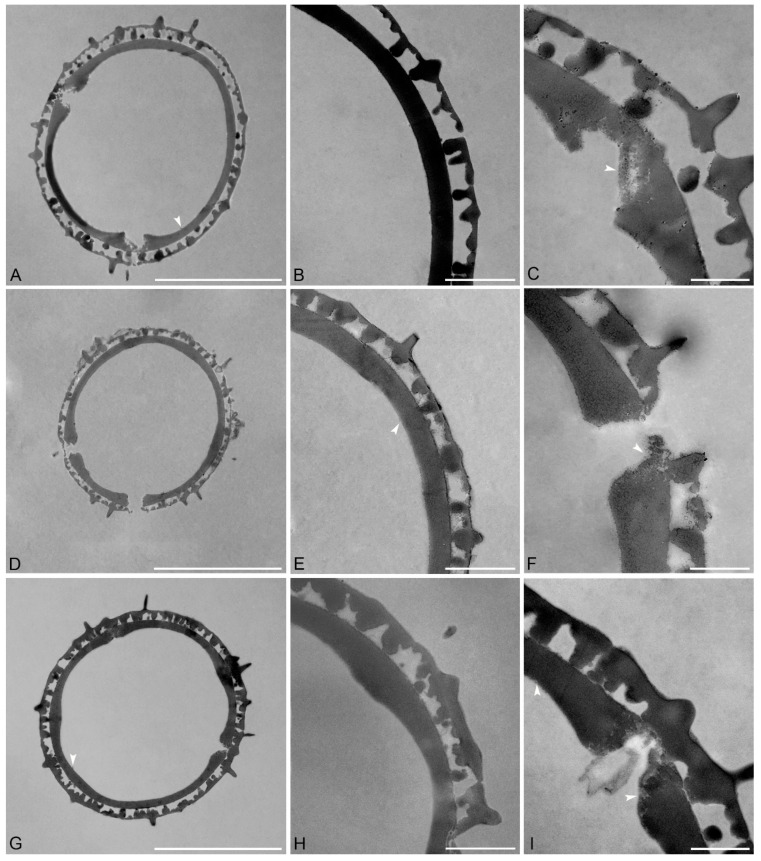
TEM micrographs of *Pseudanthus* pollen. (**A**–**C**) *Pseudanthus orbicularis* (MEL2013477). (**A**) Overview, whole cross-section. (**B**) Interapertural area. (**C**) Apertural area. (**D**–**F**) *Pseudanthus orientalis* (MEL281781). (**D**) Overview, whole cross-section. (**E**) Interapertural area. (**F**) Apertural area. (**G**–**I**) *Pseudanthus ovalifolius* (MEL2425430). (**G)** Overview, whole cross-section. (**H**) Interapertural area. (**I**) Apertural area. White arrows indicate the endexine. Scale bars—10 µm (**A**,**D**,**G**), 2 µm (**B**,**E**,**H**), and 1 µm (**C**,**F**,**I**).

Species: *Pseudanthus orbicularis* (Müll.Arg.) Halford & R.J.F.Hend. (Figure 3A–C and Table 2).

Description: Pollen diameter incl. echini 20–23.1 µm, ektexine incl. echini 2.5–2.8 µm thick, ektexine excl. echini 1.3–1.9 µm thick; ektexine composed of a columellate-tectate pollen wall with supratectal echini, echini 0.7–1.2 µm in height and 0.4–0.7 µm wide at base; eutectum perforate, tectum 0.2–0.3 µm thick; columellate infratectum 0.5–0.6 µm thick; compact-continuous foot layer, 0.6–0.8 µm thick at interapertural areas, foot layer thickened around apertures, up to 1.1–1.4 µm thick, with frayed granular ends; very thin, spongy, and discontinuous endexine (0–0.1 µm) present below foot layer, also in apertural regions and closing the apertures.

Remarks: *Pseudanthus orbicularis* has the largest pollen among the investigated species with grains reaching max. 23.1 µm in diameter. The pollen of this species is much larger than that of *P*. *divaricatissimus* (18.8–19.9 µm) and *P. ligulatus* subsp. *volcanicus* (16.2–16.8 µm) but only slightly larger than pollen of *P. micranthus* (20.3–21.1 µm). The ektexine incl. echini in pollen of *P. orbicularis* is of similar thickness but with a thinner foot layer compared to pollen of *P*. *divaricatissimus*.

Species: *Pseudanthus orientalis* F.Muell. (Figure 3D–F and Table 2).

Description: Pollen diameter incl. echini 15–16.3 µm, ektexine incl. echini 1.9–2.3 µm thick, ektexine excl. echini 1.1–1.4 µm thick; ektexine composed of a columellate-tectate pollen wall with supratectal echini, echini 0.8–1.1 µm in height and 0.5–0.7 µm wide at base; eutectum perforate, tectum 0.1–0.2 µm thick; columellate infratectum, 0.3–0.6 µm thick; compact-continuous foot layer, 0.5–0.7 µm thick at interapertural areas, foot layer thickened around apertures, up to 1–1.1 µm thick, with frayed, lamellar to granular ends; very thin endexine (0–0.05 µm) present below foot layer.

Remarks: The pollen of *P. orientalis* is smaller than pollen of *P. ligulatus* subsp. *volcanicus*, but the thickness of the ektexine is comparable.

Species: *Pseudanthus ovalifolius* F.Muell. (Figure 3G–I and Table 2).

Description: Pollen diameter incl. echini 19.7–20.3 µm, ektexine incl. echini 2.1–3 µm thick, ektexine excl. echini 1.5–1.8 µm thick; ektexine composed of a columellate-tectate pollen wall with supratectal echini, echini 1.4–1.5 µm in height and 0.5–0.7 µm wide at base; eutectum perforate, tectum 0.2–0.5 µm thick; columellate infratectum, 0.6–0.7 µm thick; compact-continuous foot layer, 0.6–0.7 µm thick at interapertural areas, foot layer thickened around apertures, up to 1.2–1.3 µm thick, with frayed, lamellar to granular ends; very thin and discontinuous (nearly non-detectable) endexine present below foot layer, also in aperture regions.

Remarks: The pollen of *P. ovalifolius* along with pollen from *P. micranthus* (20.3–21.1 µm) and *P. orbicularis* (20–23.1 µm) is among the largest pollen produced within the genus. The ektexine in pollen of *P. ovalifolius* is thinner than the ektexine in pollen of *P. micranthus*, but it is thicker than the ektexine in pollen of *P. orbicularis.* The echini in *P. ovalifolius* pollen are taller compared to echini in pollen of *P. ligulatus* subsp. *volcanicus*, *P. micranthus*, *P. orbicularis*, and *P. orientalis*.

**Figure 4:**
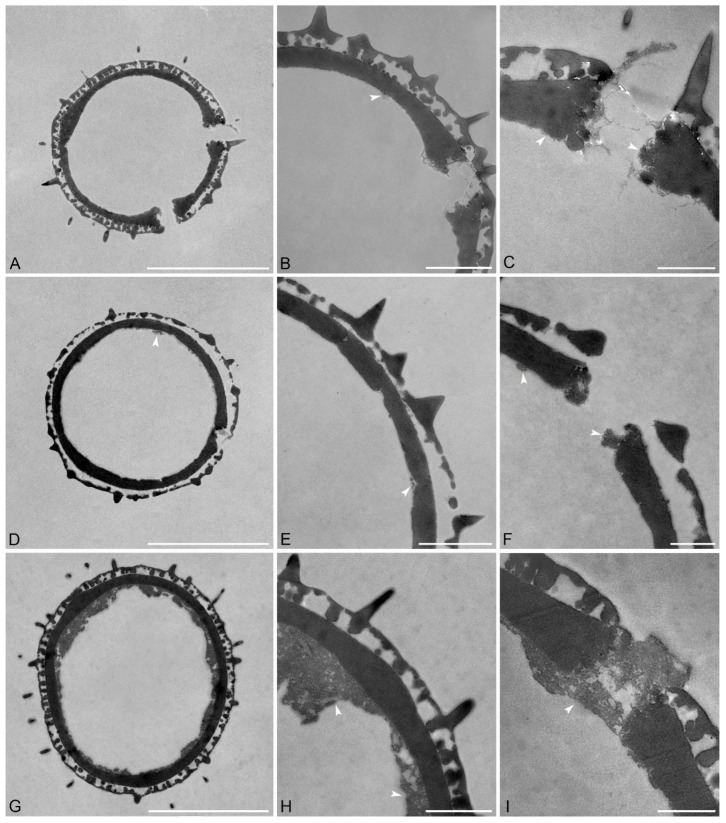
TEM micrographs of *Pseudanthus* pollen. (**A**–**C**) *Pseudanthus pauciflorus* subsp. *arenicola* (MEL2269606). (**A**) Overview, whole cross-section. (**B**) Interapertural area. (**C**) Apertural area. (**D**–**F**) *Pseudanthus pauciflorus* subsp. *pauciflorus* (MEL1596938). (**D**) Overview, whole cross-section. (**E**) Interapertural area. (**F**) Apertural area. (**G**–**I**) *Pseudanthus pimeleoides* (MEL283969). (**G**) Overview, whole cross-section. (**H**) Interapertural area. (**I**) Apertural area. White arrows indicate the endexine. Scale bars—10 µm (**A**,**D**,**G**), 2 µm (**B**,**E**,**H**), and 1 µm (**C**,**F**,**I**).

Species: *Pseudanthus pauciflorus* subsp. *arenicola* Halford & R.J.F.Hend. (Figure 4A–C and Table 2).

Description: Pollen diameter incl. echini 14.3–16.5 µm, ektexine incl. echini 1.6–2 µm thick, ektexine excl. echini 1.2–1.3 µm thick; ektexine composed of a columellate-tectate pollen wall with supratectal echini, echini 0.7–0.9 µm in height and 0.4–0.7 µm wide at base; eutectum perforate, tectum 0.1–0.3 µm thick; columellate infratectum, 0.3–0.5 µm thick; compact-continuous foot layer, 0.5–0.8 µm thick at interapertural areas, foot layer thickened around apertures, up to 0.9–1.1 µm thick, with frayed, granular ends; very thin and discontinuous endexine, 0–0.1 µm thick.

Remarks: Pollen of *P. pauciflorus* subsp. *arenicola* has the smallest diameter incl. echini reaching a minimum of 14.3 µm within the genus. Fittingly, the thickness of the ektexine incl. echini is the thinnest among pollen from all the investigated *Pseudanthus* species.

Species: *Pseudanthus pauciflorus* subsp. *pauciflorus* (Figure 4D–F and Table 2).

Description: Pollen diameter incl. echini 18.1–18.7 µm, ektexine incl echini 1.8–2.2 µm thick, ektexine excl. echini 1.2–1.4 µm thick; ektexine composed of a columellate-tectate pollen wall with supratectal echini, echini 0.9–1 µm in height and 0.5–0.6 µm wide at base; eutectum perforate, tectum 0.2–0.3 µm thick; columellate infratectum, 0.4–0.5 µm thick; compact-continuous foot layer, 0.7–0.8 µm thick at interapertural areas, foot layer thickened around aperture, up to 0.9 µm thick, partly with frayed at the ends; very thin, spongy, and discontinuous endexine present below foot layer, 0–0.1 µm thick, also in apertural regions.

Remarks: The *P. pauciflorus* subsp. *pauciflorus* pollen grains are larger than the ones in the subsp. *arenicola*. Still, the thickness of the ektexine incl. echini is only slightly thicker than that in pollen of *P. pauciflorus* subsp. *arenicola*.

Species: *Pseudanthus pimeleoides* Sieber ex Spreng. (Figure 4G–I and Table 2).

Description: Pollen diameter incl. echini 18.8–20.1 µm, ektexine incl. echini 2–2.4 µm thick, ektexine excl. echini 1.4–1.5 µm thick; ektexine composed of a columellate-tectate pollen wall with supratectal echini, echini 0.8–1.6 µm in height and 0.5–0.6 µm wide at base; eutectum perforate, tectum 0.1–0.2 µm thick; columellate infratectum, 0.5–0.6 µm thick; compact-continuous foot layer, 0.7–0.8 µm thick at interapertural areas, foot layer thickened around apertures, 1.1–1.2 µm thick, with frayed and granular ends; spongy and discontinuous endexine, 0.1–2 µm thick, also in apertural regions, closing the apertures.

Remarks: Pollen of *P. pimeleoides* as well as that from *P. divaricatissimus* both have a thick and discontinuous endexine compared to pollen from the other *Pseudanthus* species.

### 2.2. Updated Genus-Based Pollen Description—Combined LM, SEM, and TEM

To provide a comprehensive overview of the morphology and ultrastructure of *Pseudanthus* pollen, the TEM analyses from this study are here combined with previous LM- (acetolyzed, glycerine) and SEM (acetolyzed, air dried)-based morphological features from Till et al. [7]. The following genus description now covers all pollen morphological and ultrastructural features observed from eight out of the nine accepted *Pseudanthus* species.

Description: Pollen, monad, P/E ratio ± isodiametric, shape ± spherical (LM, SEM); diameter incl. echini 14.2–27.1 μm in LM, 12.6–24.2 μm in SEM, 14.3–23.1 µm in TEM; pantoporate, 5–19 pori (LM), aperture outline circular to elliptic, aperture margin can be clear to unclear or diffuse (LM, SEM), pori diameter 0.7–2.6 µm in LM, 0.6–1.6 µm in SEM; exine 1.2–2.8 µm thick in LM, nexine thicker than sexine, nexine 0.7–1.6 µm thick, sexine 0.5–1.3 µm thick (LM); pollen wall tectate (SEM, TEM), ektexine incl. echini 1.6–3.9 µm thick (TEM), ektexine excl. echini 1–2.3 µm (TEM); sculpture echinate (LM, SEM, TEM) and psilate, perforate, and nanoverrucate to granular between echini (SEM, TEM); 4.5–15.5 echini per 25 µm^2^ pollen surface, echini 0.5–1.8 µm (SEM) and 0.6–1.9 µm (TEM) in height, and 0.3–0.9 µm (SEM) and 0.4–0.9 µm (TEM) wide at the base; eutectum perforate (SEM, TEM), tectum 0.1–0.5 µm thick (TEM); compact-continuous foot layer, 0.5–1.1 µm thick (TEM); columellate infratectum, 0.3–0.9 µm thick (TEM); foot layer thickened around apertures, up to 0.9–2 µm thick, foot layer with frayed, lamellar to granular ends (TEM); endexine varying, often very thin, can be spongy, discontinuous, between 0 and 2 µm thick, also present in apertural regions (TEM), closing the apertures (SEM, TEM); aperture membrane with verrucate, granulate to accumulated granulate, and nanoareolate sculpture (SEM).

Remarks: In both LM and SEM, *Pseudanthus* pollen is isodiametric to slightly oblate with a diameter of 12.6–27.1 μm, depending on the preparation method and if it is observed with LM, SEM, or TEM. In LM, the pollen is clearly pantoporate, but this aperture configuration is harder to identify in SEM and especially with TEM. In both LM and SEM, the pori are circular to elliptic in outline. While the exine can be differentiated into nexine and sexine in LM, the ultrastructure observed with TEM differentiates the pollen wall into a thin endexine and massive and highly structured ektexine. The latter comprises a foot layer, infratectum, tectum, and supratectal elements. In many of the investigated pollen grains, the endexine was hard to detect, often appearing very thin (c. 0.05 µm), and it did not stain with potassium permanganate. Most notable was the foot layer, as it was always markedly thick and continuous, which is very characteristic for pollen of this genus. Also of interest are the perforations, observed both with SEM and via the eutectate tectum with TEM.

## 3. Discussion

### 3.1. Ultrastructural Variability in Pseudanthus Pollen

*Pseudanthus orbicularis* pollen has the widest diameter, 20–23.1 µm, while the shortest diameter occurs in *P. pauciflorus* subsp. *arenicola* pollen. Fittingly, the ektexine of *P. pauciflorus* subsp. *arenicola* pollen is the thinnest (1.6–2 µm.). *Pseudanthus micranthus* pollen has the thickest ektexine incl. echini with 2.9–3.9 µm. Endexine thickness in pollen from six out of the eight species is similar with very thin, discontinuous layers below the foot layer. The endexine is most easily observed in the pollen of *P. divaricatissimus* and *P. pimeleoides*, where it reaches up to 1.2 µm in thickness in the pollen of *P. divaricatissimus* and 2 µm in the pollen of *P. pimeleoides*. In the pollen from these two taxa, a spongy discontinuous layer occurs under the foot layer that can be observed filling the apertures (Figure 4I). The configuration of this layer can vary between pollen from different species and at an intraspecific level. For example, in different *P. divaricatissimus* pollen, the endexine occurred either as a thin strip below the foot layer or as a thick endexine layer (Figure 4A–C). In *P. ligulatus* subsp. *volcanicus* pollen (Figure 2D,F) parts of the endexine, thin bands, were detached from the foot layer. In the interapertural areas of pollen from most species, the foot layer is between 0.5 and 0.8 µm thick, and only *P. divaricatissimus* has pollen with a thicker foot layer that is between 0.8 and 1.1 µm thick. The foot layer becomes thicker around the apertures, sometimes up to twice the thickness of that observed at interapertural areas. For example, in the pollen of *P. divaricatissimus*, the interapertural foot layer is between 0.8 and 1.1 µm thick and the apertural foot layer is 1.6–2 µm thick. Pollen of *P. pauciflorus* subsp. *pauciflorus* (Figure 4D–F) has the thinnest foot layer around the apertures, with the interapertural foot layer being 0.7–0.8 µm thick and that around the apertures up to 0.9 µm thick. The infratectum is columellate in the pollen from all species investigated, but the fewest columellae occur in the pollen of *P. pauciflorus* subsp. *pauciflorus*. The tectum is between 0.1 and 0.5 µm thick, with the pollen of *P. divaricatissimus* (0.2–0.5 µm) and *P. ovalifolius* (0.2–0.5 µm) having the widest range and the thickest tecta. The highest echini occur in pollen of *P. divaricatissimus* (1.3–1.9 µm), *P. ovalifolius* (1.4–1.5 µm), and *P. pimeleoides* (0.8–1.6 µm). However, the measured height of the echini also depends on the cutting angle when the sections were produced and should be considered with caution.

### 3.2. Cluster Grouping of Pseudanthus Species Based on Pollen Characteristics

Based on the combined ultrastructural and morphological characteristics (LM, SEM, TEM), the investigated *Pseudanthus* species are grouped into three morphologically coherent clusters. The main traits used for clustering include pollen size, exine and echini thickness, and foot layer and endexine structure, which are particularly diagnostic for this genus. These groupings help highlight both interspecific variation and potential phylogenetic or ecological trends within *Pseudanthus*. The first cluster is characterized by large pollen with a relatively thick ektexine (both incl. and excl. echini), and more robust exine features including taller echini and consistently thickened foot layers. They likely represent a morphologically advanced group within the genus, possibly adapted to more exposed environments requiring more robust pollen walls. *Pseudanthus* species included in this cluster are *P. micranthus* (largest ektexine up to 3.9 µm, incl. echini, large diameter 20.3–21.1 µm, thickest ektexine excl. echini up to 2.3 µm), *P. orbicularis* (largest pollen up to 23.1 µm, relatively thick ektexine incl. echini 2.5–2.8 µm, thinner foot layer), and *P. ovalifolius* (large pollen 19.7–20.3 µm, tall echini 1.4–1.5 µm, thick exine, very thin/discontinuous endexine). The second cluster is characterized by medium-sized pollen with a thick endexine and very tall echini and/or a thick, spongy endexine. This may be interpreted as an intermediate group showing transitional pollen characteristics between larger and smaller pollen types, with a specialization in endexine structure. *Pseudanthus* species included in this cluster are *P. divaricatissimus* (medium size 18.8–19.9 µm, tallest echini 1.3–1.9 µm, spongy/discontinuous endexine), *P. pimeleoides* (similar size, thick and spongy endexine up to 2 µm, also echini quite tall up to 1.6 µm), and *P. pauciflorus subsp. pauciflorus* (slightly smaller 18.1–18.7 µm, similar exine thickness and echini height, but foot layer slightly less thickened). The third cluster comprises species with smaller pollen grains with a thinner ektexine overall, shorter echini, and a very thin or nearly absent endexine. This may be interpreted as a more basal or specialized group with reduced pollen walls, likely reflecting adaptation to specific ecological niches or reduced environmental stressors. *Pseudanthus* species included in this cluster are *P. ligulatus* subsp. *volcanicus* (small size 16.2–16.8 µm, thinnest infratectum and endexine ~0.025 µm, short echini), *P. orientalis* (smallest pollen diameter 15–16.3 µm, thin exine and endexine ~0.025 µm, short echini), and *P. pauciflorus* subsp. *arenicola* (smallest size overall 14.3–16.5 µm, thinnest ektexine incl. echini 1.6–2 µm, echini also short).

### 3.3. The Ultrastructure of Pseudanthus Pollen Compared to That of Other Picrodendraceae

The Picrodendraceae currently includes 25 genera and approximately 98 species [2]. Pollen morphology of Picrodendraceae has been investigated by Punt [5], Köhler [8], Martin [14], Hayden et al. [15], Levin and Simpson [6], Simpson and Levin [9], Grímsson et al. [16], and Till et al. [7]. However, ultrastructural analysis of Picrodendraceae pollen has been limited, with only Levin and Simpson [6], Simpson and Levin [9,15], and Hayden et al. [15] conducting such studies. Table 3 summarizes the ultrastructural traits of 22 Picrodendraceae genera investigated by Levin and Simpson [6] and Simpson and Levin [9], based on living pollen fixed in glutaraldehyde and osmium tetroxide. The data from Hayden et al. [15] were excluded due to the lack of precise scale markers. For comparability, pollen morphological traits such as the P/E ratio, shape, diameter, and aperture configuration, extracted from Köhler [8], Martin [14], Levin and Simpson [6], and Grímsson et al. [16], have been incorporated (Table 3). Based on these findings, the P/E ratio of Picrodendraceae pollen is isodiametric to oblate and the shape varies between spherical and oblate spheroidal. Among the taxa investigated, 15 genera produce stephanoporate pollen, while 7 genera exhibit pantoporate pollen. Among pantoporate Picrodendraceae pollen, *Micrantheum*, *Neoroepera*, *Scagea,* and *Stachystemon* show similarities to *Pseudanthus* in their pollen wall structures. Nevertheless, there are some differences. The thick, continuous foot layer in *Micrantheum* measures up to 1.73 µm (Table 3) and is twice as thick as in *Pseudanthus* (ranging from 0.5 to 1.1 µm) (Table 2 and Table 3). *Neoroepera* exhibits thicker columellae than *Pseudanthus*, with a thin, perforated, semi- to eutectate tectum. In *Stachystemon*, the foot layer is thinner, and the echini are similar in size compared to those of *Pseudanthus*, with a very thin tectum. *Scagea* pollen is characterized by a thick, continuous foot layer, a thin tectum, and a notably thin infratectum with columellae. The ultrastructural traits of *Kairothamus* pollen seem to overlap with that of *Pseudanthus* (Table 3). Pollen of both taxa exhibit a thick continuous foot layer that is c. 0.61 µm thick in *Kairothamnus* and 0.5–1.1 µm thick in *Pseudanthus* (Table 2 and Table 3). The pollen walls in both taxa also have a columellate infratectum and a thin and perforated eutectum. In both taxa, the foot layer is thickened around apertures and the thin endexine is extending across the aperture. According to Levin and Simpson [6], the foot layer is absent in *Kairothamnus* and the endexine is slightly thicker at the apertures.

### 3.4. Misinterpretations of Foot Layer and Endexine in Picrodendraceae

Misinterpretations of the foot layer and endexine in Picodendraceae pollen have been detected during this study. Therefore, all pollen wall layers previously described/illustrated have been critically revised and summarized in Table 3. The configuration of the foot layer in Picrodendraceae pollen is variable: some species produce pollen seemingly without a foot layer, while other produce pollen with a thin or thick foot layer (Table 3). Picrodendracae taxa seemingly lacking a foot layer are *Aristogeitonia monophylla Airy Shaw*, *Longetia buxoides* Baill., *Mischodon zeylanicus* Thwaites, *Oldfieldia africana* Benth. & Hook.f., and *Piranhea* Baill., but more TEM micrographs are needed to verify this (Table 3). In some cases, the foot layer was obviously misinterpreted and reported to be absent by Levin and Simpson [6] and Simpson and Levin [9], such as for *Androstachys johnsonii* Prain, *Choriceras majus* Airy Shaw, *Dissiliaria baloghioides* F.Muell. ex Baill., *Parodiodendron marginivillosum* (Speg.) Hunz., *Petalostigma quadriloculare* F.Muell., *Piranhea trifoliolata* Baill. and *Piranhea mexicana* (Standl.) Radcl.-Sm., *Stachyandra merana* (Airy Shaw) J.-F.Leroy ex Radcl.-Sm. and *Stachyandra rufibarbis* (Airy Shaw) Radcl.-Sm., and *Whyanbeelia terrae-reginae* Airy Shaw & B.Hyland (Table 3). A thin foot layer, which may be continuous or more-or-less continuous and range between 0.01 and 0.35 µm in thickness, occurs in pollen of *Androstachys johnsonii* Prain, *Austrobuxus carunculatus* (Baill.) Airy Shaw, *Choriceras majus* Airy Shaw, *Dissiliaria baloghioides* F.Muell. ex Baill., *Hyaenanche globosa* (Gaertn.) Lamb. & Vah, *Neoroepera buxifolia* Müll.Arg., *Parodiodendron marginivillosum* (Speg.) Hunz., *Petalostigma quadriloculare* F.Muell., *Picrodendron baccatum* (L.) Krug & Urb., *Podocalyx loranthoides* Klotzsch, *Stachyandra merana* (Airy Shaw) J.-F.Leroy ex Radcl.-Sm., *Stachyandra rufibarbis* (Airy Shaw) Radcl.-Sm., *Tetracoccus dioicus* Parry, *Voatamalo eugenioides* Capuron ex Bosser, and *Whyanbeelia terrae-reginae* Airy Shaw & B.Hyland (Table 3). Picodendraceae characterized by a thick foot layer, which is mostly continuous or more-or-less continuous and ranges between 0.5 and 1.73 µm in thickness, are *Kairothamnus phyllanthoides* (Airy Shaw) Airy Shaw, *Micrantheum hexandrum* Hook.f., *Neoroepera banksii* Benth., *Pseudanthus divaricatissimus* (Müll.Arg.) Benth., *Scagea oligostemon* (Guillaumin) McPherson, and *Stachystemon polyandrus* (F.Muell.) Benth. In the two described *Neoroepera* species, the foot layer varies significantly from thick, continuous (1.0 µm), and thicker at the apertures in *N. banksii* to thin and discontinuous (0.05 µm) without aperture thickenings in *N. buxifolia*. Differences in these two species are also found in the infratectum and tectum thickness, and the presence or absence of an endexine. Cases where the foot layer has been interpreted as endexine have been found in Levin and Simpson [6] for *Androstachys johnsonii* Prain., *Choriceras majus* Airy Shaw, *Dissiliaria baloghioides* F.Muell. ex Baill., *Parodiodendron marginivillosum* (Speg.) Hunz., *Petalostigma quadriloculare* F.Muell., *Stachyandra merana* (Airy Shaw) J.-F.Leroy ex Radcl.-Sm., *Stachyandra rufibarbis* (Airy Shaw) Radcl.-Sm., and *Whyanbeelia terrae-reginae* Airy Shaw & B.Hyland (Table 3). In other cases, it remains unclear based on the available TEM micrographs whether an endexine is present or absent, such as for *Stachystemon polyandrus* (F.Muell.) Benth., and contrary to earlier descriptions by Simpson and Levin [9], *Petalostigma quadriloculare* F.Muell. is obviously lacking an endexine layer. These misinterpretations also affect the interpretation of ektexine thickenings around apertures, one of the main characteristics in Picrodendraceae pollen. Whether the foot layer or the endexine are thickened towards the aperture and whether the endexine extends across the aperture has been revised based on previously illustrated Picrodendraceae pollen (extracted from Levin and Simpson [6]; Simpson and Levin [9], and Hayden et al. [15]) and summarized in Table 3. Still, further studies on the pollen wall ultrastructure of Picrodendraceae genera and species are needed to clarify discrepancies and uncertainties in the configuration of the foot layer and endexine (Table 3). Since TEM technology, including high resolution imaging techniques and refined sample preparation methods have improved dramatically in the last years, a renewed ultrastructural investigation can make a significant contribution to solving these issues [10].

### 3.5. Pseudanthus Pollen Compared to That from Euphorbiaceae s. l., Crotonoideae (Euphorbiaceae s. str.) and Phyllanthaceae (Former Subfamily of Euphorbiaceae s. str.)

*Pseudanthus* was formerly placed in Euphorbiaceae [3]. Pollen morphology has long played a role in the systematics of Euphorbiaceae s.l. Pollen of Euphorbiaceae s.l. was originally studied by Punt [5] with LM. At that time, the author divided the plant taxa into subfamilies Phyllanthoideae and Crotonoideae, describing a wide range of different configuration types. The Phyllanthoideae, a former subfamily of Euphorbiaceae s. l., are now partly placed in the Phyllanthaceae, which comprises 59 genera and about 1285 species [2]. At the genus level, pollen grains of the Euphorbiaceae tend to be relatively uniform. However, in large genera, such as *Tragia* Plum. ex L. (subfamily Acalyphoideae Beilschm.) and *Phyllanthus* L. (Phyllanthaceae Martinov), genus-specific differences in pollen morphology/ultrastructure are observed. Pollen of the former Phyllanthoideae has been categorized into six configuration types. The pollen grains are frequently tricolporate or stephanocolporate, occasionally periporate or inaperturate, and their surfaces can be tectate or atectate, and the sculpture reticulate, echinate, or psilate [5]. A notable example is the *Phyllanthus nutans* pollen type, which is pantoporate, similar to *Pseudanthus*. However, the pollen surface is uniquely divided into pentagons or hexagons, with pori situated at the corners, a pattern distinct from the randomly distributed pori in *Pseudanthus*. The pollen of *Pseudanthus* falls into the *Stachystemon* type within the *Aristogeitonia* configuration, which fits well with the plant morphological characteristics [19]. Pollen grains assigned to this type/configuration are either pantoporate or inaperturate, spherical to spheroidal, and are tectate with an echinate sculpture [5]. More recently, Levin and Simpson [6] analyzed pollen from 10 genera of Phyllanthoideae/Phyllanthaceae. Their findings are consistent with Punt’s study from 1962 [5]. The pollen of these genera (*Amanoa* Aubl., *Antidesma* L., *Hymenocardia* Wall. ex Lindl., etc.) is prolate (spheroidal), prolate to isodiametric (spheroidal to spherical), oblate to isodiametric (oblate-spheroidal to spherical), and 3-colpate. Interestingly, only *Phyllanthus* includes species with pollen that varies from tricolpate to 60-pantoporate [6]. In contrast, the Crotonoideae (Euphorbiaceae s.str.) show even greater diversity in aperture and ornamentation types, with 12 described distinguishable pollen configurations [5]. These include variations in aperture configuration, such as inaperturate, triporate, tricolporate, tricolpate, stephanoporate, stephanocolporate, and stephanocolpate. However, none of these pollen configurations comprise pantoporate pollen types, making it easy to differentiate them from *Pseudanthus.*

### 3.6. Taxonomic Implications

The pantoporate pollen type is a key feature that clearly separates *Pseudanthus* (and other Picodendraceae) from Euphorbiaceae s.str. This study not only supports its placement within Picrodendraceae but also distinguishes it from morphologically similar taxa within the family, as well as in Euphorbiaceae and Phyllanthaceae, reinforcing the palynological boundaries among these closely related families. The presence of pantoporate pollen alone distinguishes *Pseudanthus* from Crotonoideae, while the combination of an echinate sculpture, tectate structure, and foot layer morphology clearly separates it from morphologically similar genera in Phyllanthaceae. The palynological distinctiveness of Picrodendraceae validates its taxonomic separation from Euphorbiaceae s.str. and Phyllanthaceae.

## 4. Material and Methods

### 4.1. Origin of Plant Material

Pollen samples from eight of the nine existing *Pseudanthus* species [1] were collected from anthetic flowers stored in the National Herbarium of Victoria (MEL) in Melbourne, Australia (Table 1).

### 4.2. Pollen Preparation for Transmission Electron Microscopy and Storage

Flowers were examined using a binocular microscope and anthers were extracted for pollen analysis. The anthers were placed into drops of acetolysis mixture (9 parts 99% acetic anhydride and 1 part 96% sulfuric acid) on an LM glass slide and processed following the “fast acetolysis method” described by Halbritter et al. [13]. The anthers were then crushed with a dissection needle to release pollen into the liquid. Following this, the slides were repeatedly heated over a candle flame for 2 to 5 s to speed up the maceration process and facilitate the release of cell contents from pollen grains. Following acetolysis, the pollen grains were gathered at the edge of the acetolysis drop using a micromanipulator (nasal hair attached to a dissection needle) and a light microscope with an extended working distance. In the next step, a small drop of glycerine was placed on a clean glass slide, and using the micromanipulator, the pollen grains were transferred from the acetolysis into the glycerine drop. The TEM preparation and ultramicrotomy followed the protocol by Ulrich and Grímsson [20]. Single pollen grains from each species were transferred from the glycerine drops into embedding molds filled with a mixture of three to four drops of epoxy resin and three to four drops of acetone. After adding pollen grains into the filled mold, an extra drop of acetone was added to the resin–acetone mixture to clean glycerine from the pollen surfaces. One to three pollen grains were transferred into each mold. The embedding forms were then placed into an oven at 70 degrees for about 12 h to accelerate polymerization. Following polymerization, the specimen blocks were taken out of the molds, labelled, and mounted on a cylinder-shaped resin block. The specimen blocks were then fixed in an ultramicrotome block holder and trimmed under the binocular with a razor blade to obtain a small block face in trapezoid form. For sectioning, a LEICA EM UC6 ultramicrotome (LEICA, Wetzlar, Germany) and a DiATOME Ultra 45° diamond knife (DiATOME, Biel, Switzerland) were used to make ultra-thin sections (70–80 nm). The sections were stretched using xylene and transferred with a suitable loop onto formvar film-coated copper slot grids and stored in a grid box. To reveal the lipidic endexine, one or two ultra-thin sections were stained with a 1% aqueous solution of potassium permanganate (KMnO_4_), which stains phospholipid–protein complexes. This process involved immersing the sections in a drop of the staining solution for 5 min, followed by rinsing in three drops of deionized water for 5 min each [21]. The sections were then observed with a Zeiss EM 900N transmission electron microscope (Zeiss, Jena, Germany) at 80 kV. Overview and close-up micrographs of pollen grains were taken at high magnification between 12,000 and 20,000 using a slow-scan CCD camera for TEM (2K-Wide-angle, TRS, Moorenweis, Germany), a CCD digital camera controller (Moorenweis, Germany), and image SP software (IspSoftware64, Moorenweis, Germany). The ultrathin sections produced during this study are stored in the collections of the Division of Structural and Functional Botany, Department of Botany and Biodiversity Research, University of Vienna, Vienna, Austria.

The pollen prepared for this study were compared to results from previous works using different preparation methods. It should be noted that preparation methods can affect the outcome observed with LM, SEM, and TEM. This especially refers to the measurements of particular pollen features, including grain diameter.

### 4.3. Descriptions and Measurements Based on TEM Micrographs

Pollen measurements were made on TEM micrographs in Adobe Photoshop (Version 25.1.0). Measurements of pollen grain diameter (a), ektexine incl. echini (b), ektexine excl. echini (c), foot layer (d), infratectum (e), tectum (f), foot layer at aperture (g), aperture diameter (h), echinus height (i), echinus base (j), and endexine (k) were obtained (Figure 1). For each species, one or two pollen grains were measured (Table 2), and two measurements (shortest/thinnest vs. longest/thickest etc.) were acquired for each ultrastructural trait.

*Remark*: An attempt was made to section pollen grains close to the equatorial plane, providing sections through one or more apertures, but this varied between samples. It should be noted that the position where the section was made, as well as the respective incision through the pollen wall (e.g., lateral, tangential, diagonal) can affect measurements of the different features. For instance, echini not sectioned through their central axis may appear shorter or broader, and measurements may lead to overestimation of wall thickness. To account for this, multiple measurements were taken for each feature, and the minimum and maximum values were used in the analysis, as these are more likely to reflect the true dimensions. Nonetheless, some variability due to sectioning angle cannot be entirely excluded, and measurements were interpreted with appropriate caution.

## 5. Conclusions

Combined LM, SEM, and TEM investigations of angiosperm pollen are often the only way to clearly differentiate between the pollen of closely related taxa. For Picrodendraceae pollen, aperture arrangement (observed with LM) in combination with the configuration of the foot layer and endexine (observed with TEM) can segregate pollen from different species, species groups, or genera within the family. Importantly, our observations reveal inconsistencies in previous interpretations of foot layer and endexine structures, particularly in taxa where earlier studies lacked resolution or misidentified pollen wall layers. This study provides revised interpretations and standardized terminology based on consistent morphological criteria. Previous LM and SEM work by Till et al. [7] showed that it was possible to distinguish *Pseudanthus* pollen from all other Picrodendraceae pollen except that of *Kairothamnus, Scagea*, and *Stachystemon*, based on suites of LM and SEM features. With added ultrastructural traits from this study, the differences between *Pseudanthus* and pollen from most other Picrodendraceae genera have become clearer. More importantly, it also enables segregation between *Pseudanthus* pollen and that from both *Scagea* and *Stachystemon*. Based on combined LM, SEM, and TEM analyses, *Kairothamnus* pollen appears most like that of *Pseudanthus*, but further SEM studies on the sculpture of *Kairothamnus* pollen are needed to fully compare pollen from these two genera. The insights gained from this research not only expand our knowledge of *Pseudanthus* but also provide valuable data for future comparative studies on Picrodendraceae and pollen from other related plant groups. This research also lays the ground for future discoveries of *Pseudanthus* pollen from the fossil palynological record. Until now, *Pseudanthus* pollen has never been reported from any fossil assemblage. This might be the result of the previous lack of descriptions and micrographs (LM, SEM, TEM) depicting extant *Pseudanthus* pollen and the lack of the means to differentiate pollen of this genus from other similar angiosperm pollen. Further studies are still needed to resolve remaining uncertainties, particularly in taxa where important characteristics such as the presence and thickness of individual wall layers remain ambiguous.

## Figures and Tables

**Table 1:** Herbarium specimens used for this study.

Species	Accession Number	Link to Virtual Herbarium
*P. divaricatissimus* (Müll.Arg) Benth	MEL2291167	https://avh.ala.org.au/occurrences/7a9d7870-a239-454e-994f-04a66822f0ba
*P. ligulatus* subsp. *volcanicus* Halford & R.J.F.Hend	MEL1526549	https://avh.ala.org.au/occurrences/a61c17fa-57ec-4350-b69c-f25470239643
*P. micranthus* Benth.	MEL625058	N.A.
*P. orbicularis* (Müll.Arg.) Halford & R.J.F.Hend	MEL2013477	https://avh.ala.org.au/occurrences/6020b741-6133-4fac-ac2c-fb7be8bf0b61
*P. orientalis* F.Muell.	MEL281781	N.A.
*P. ovalifolius* F.Muell.	MEL2425430	https://avh.ala.org.au/occurrences/656a8565-df43-444f-9d41-fa5f12d12914
*P. pauciflorus* subsp. *arenicola*	MEL2269606	https://avh.ala.org.au/occurrences/68fa2391-6bab-4f3d-a45a-7ec6c5c68041
*P. pauciflorus* subsp. *pauciflorus*	MEL1596938	https://avh.ala.org.au/occurrences/0dd4ce9b-ab33-4fdd-b162-64ca8b6a8037
*P. pimeleoides* Sieber ex Spreng	MEL283969	N.A.

**Table 2:** Pollen ultrastructure of *Pseudanthus* and other features observed with TEM.

	*P. divaricatissimus*	*P. ligulatus* subsp. *volcanicus*	*P. micranthus*	*P. orbicularis*	*P. orientalis*	*P. ovalifolius*	*P. pauciflorus* subsp. *arenicola*	*P. pauciflorus* subsp. *pauciflorus*	*P. pimeleoides*	*Pseudanthus* (Genus Range)
**P/E ratio**	Isodiametric to oblate	Isodiametric to oblate	Isodiametric to oblate	Isodiametric to oblate	Isodiametric to oblate	Isodiametric to oblate	Isodiametric to oblate	Isodiametric to oblate	Isodiametric to oblate	Isodiametric to oblate
**Shape**	Spherical to oblate spheroidal	Spherical to oblate spheroidal	Spherical to oblate spheroidal	Spherical to oblate spheroidal	Spherical to oblate spheroidal	Spherical to oblate spheroidal	Spherical to oblate spheroidal	Spherical to oblate spheroidal	Spherical to oblate spheroidal	Spherical to oblate spheroidal
**Diameter incl. echini**	18.8–19.9	16.2–16.8	20.3–21.1	20–23.1	15–16.3	19.7–20.3	14.3–16.5	18.1–18.7	18.8–20.1	14.3–23.1
**Ektexine incl. echini thickness**	2.3–3.7	2.1–2.2	2.9–3.9	2.5–2.8	1.9–2.3	2.1–3	1.6–2	1.8–2.2	2–2.4	1.6–3.9
**Ektexine excl. echini thickness**	1.5–2.1	1–1.2	1.9–2.3	1.3–1.9	1.1–1.4	1.5–1.8	1.2–1.3	1.2–1.4	1.4–1.5	1–2.3
**Endexine thickness**	0–1.2	0–0.05	0–0.05	0–0.1	0–0.05	0–0.1	0–0.1	0–0.1	0.1–2	0–2
**Endexine features**	Discontinuous, spongy; also in aperture regions, closing the apertures	Very thin	Very thin, discontinuous; also in aperture regions	Very thin, discontinuous, spongy in apertural regions, closing the apertures	Very thin	Very thin, discontinuous, nearly non-detectable; also in aperture regions	Very thin, discontinuous	Discontinuous, spongy; also in aperture regions	Discontinuous, spongy; also in aperture regions, closing the apertures	Very thin, spongy, discontinuous; also in aperture regions, closing the apertures
**Interapertural foot layer thickness**	0.8–1.1	0.5–0.6	0.8–0.9	0.6–0.8	0.5–0.7	0.6–0.7	0.5–0.8	0.7–0.8	0.7–0.8	0.5–1.1
**Thickened foot layer around apertures**	Present	Present	Present	Present	Present	Present	Present	Present	Present	Present
**Foot layer at aperture thickness**	1.6–2	1.0–1.1	1.3–1.4	1.1–1.4	1–1.1	1.2–1.3	0.9–1.1	0.9–1	1.1–1.2	0.9–2
**Foot layer features**	Thick, compact-continuous with frayed, granular ends, thickened around apertures	Thick, compact-continuous with frayed, lamellar to granular ends, thickened around apertures	Thick, compact-continuous with frayed, lamellar to granular ends, thickened around apertures	Thick, compact-continuous with frayed, granular ends, thickened around apertures	Thick, compact-continuous with frayed, lamellar to granular ends, thickened around apertures	Thick, compact-continuous with frayed, lamellar to granular ends, thickened around apertures	Thick, compact-continuous with frayed, granular ends, thickened around apertures	Thick, compact-continuous with ends that are a little bit frayed, thickened around apertures	Thick, compact-continuous with frayed, granular ends, thickened around apertures	Thick, compact-continuous with frayed, lamellar to granular ends, thickened around apertures
**Aperture type**	Porate	Porate	Porate	Porate	Porate	Porate	Porate	Porate	Porate	Porate
**Aperture diameter**	1.4–1.5	1–1.5	1.1–1.2	1.2–1.5	0.9–1	0.7–0.8	1.2–1.3	1.4–1.5	1–1.1	0.7–1.5
**Infratectum configuration**	Columellate	Columellate	Columellate	Columellate	Columellate	Columellate	Columellate	Columellate	Columellate	Columellate
**Infratectum thickness**	0.4–0.5	0.3–0.4	0.6–0.9	0.5–0.6	0.3–0.6	0.6–0.7	0.3–0.5	0.4–0.5	0.5–0.6	0.3–0.9
**Tectum configuration**	Eutectate	Eutectate	Eutectate	Eutectate	Eutectate	Eutectate	Eutectate	Eutectate	Eutectate	Eutectate
**Tectum thickness**	0.2–0.4	0.1–0.2	0.3–0.4	0.2–0.3	0.1–0.2	0.2–0.5	0.1–0.3	0.2–0.3	0.1–0.2	0.1–0.5
**Sculpture**	Echinate	Echinate	Echinate	Echinate	Echinate	Echinate	Echinate	Echinate	Echinate	Echinate
**Echinus height**	1.3–1.9	0.6–0.9	0.9–1	0.7–1.2	0.8–1.1	1.4–1.5	0.7–0.9	0.9–1	0.8–1.6	0.6–1.9
**Echinus width at base**	0.5–0.7	0.7–0.9	0.7–0.8	0.4–0.7	0.5–0.7	0.5–0.7	0.4–0.7	0.5–0.6	0.5–0.6	0.4–0.9
**Measured grains**	2	1	1	2	2	1	2	1	1	13

Note: All measurements given are in µm.

**Table 3:** Main pollen traits of Picrodendraceae genera.

	Combined LM and SEM Features of Genera						TEM Features of Investigated Species					
Genus/Species (Species Number)	P/E Ratio	Shape	Diameter	Aperture Configuration	Exine Sculpture	Echini Height	Endexine/Thickness	Foot Layer/Thickness	Infratectum/Thickness	Tectum/Thickness	Supratectal Elements	Aperture/Periapertural Thickening
*Androstachys* (1) *A. johnsonii*	Isodiametric to oblate	Sphere to spheroid	29–33 (50) (P) x 34–38 (50) (E)	Stephano(5–7)porate [1 or 2 pori can be outside equator]	Microechinate; granulate and perforate in areas between microechini	0.7–1.3	Thin compact-continuous layer, extending as thin layer across aperture/0.04	Thin, discontinuous/0.09, [discontinuous or absent ^1^]	Columellate/0.14, infratectum much thinner at aperture	Thick perforated eutectum/0.38, with microchannels	Microechini, only few	Thickenings absent, ektexine with foot layer and infratectum reduced, thin endexine layer present
*Aristogeitonia* (7) *A. monophylla*	Isodiametric to oblate	Sphere to spheroid	24–35 (P) x 25–36 (E)	Stephano(5–7)porate	Echinate; nanogemmate to nanorugulate in areas between echini	2–4.5	Thin compact-continuous layer, extending as thin layer across aperture/0.03	Very thin, discontinuous to absent	Columellate/0.12, discontinuous, infratectum and tectum as two distinct layers or merged	Thick, semi- to eutectum, palisade-like /0.44–0.63	Echini	Thickenings absent, endexine slightly thicker
*Austrobuxus* (22) *A. carunculatus*	Isodiametric to oblate	Sphere to spheroid	19–34 (P) x 19–34 (E)	Stephano(5–7)porate	Echinate; verrucate and perforate in areas between echini	3–5.5	Absent	Thin continuous/0.04	Columellate	Perforated semitectum, palisade-like/0.47	Echini	Foot layer thickened at aperture, ektexine thinner, but also extending across aperture
*Choriceras* (2) *C. majus*	Oblate	Spheroid	17.5–26 (P) x 20–28 (E)	Stephano(5–7)porate	Nanoechinate; perforate in areas between nanoechini	NO	Very thin (?) [misinterpreted as foot layer ^2^]	Very thin, continuous/0.07 [absent ^1^]	Thin columellate with thick columellae/0.27–0.33	Thick, perforated eutectum/0.43	Nanoechini, only few	Foot layer massively thickened around apertures
*Dissiliaria* (6) *D. baloghioides*	Oblate	Spheroid	36 (P) x 38 (E)	Stephano(5–6)porate	Echinate; microbaculate and perforate in areas between echini	4.1	Very thin, extending across aperture [misinterpreted as foot layer ^2^]	Very thin, more or less continuous/0.1 [absent ^1^]	Columellate/0.4–0.5	Thick, semi- to eutectate, internal tectum, palisade-like/1.0	Echini	Foot layer thickened around apertures, endexine slightly thickened, extending across aperture
*Hyaenanche* (1) *H. globosa*	Isodiametric to oblate	Sphere to spheroid	29–40 (P) x 32–42 (E)	Stephano(6–7)porate	Echinate; fossulate, perforate, and nanogemmate in areas between echini	1–2.1	Very thin and thickened towards the aperture	Thin, more or less continuous/0.27	Very thin, columellate to granular/0.12	Thick perforated eutectum/0.47	Echini	Endexine thickened towards the aperture
*Kairothamnus* (1) *K. phyllanthoides*	Isodiametric	Sphere	26 (D)	Panto(10–12)porate	Echinate; foveolate to perforate and granulate in areas between echini	1.8	Thin and continuous, extending as thin layer across aperture	Thick, continuous/0.61	Columellate/0.26	Thin, perforated eutectum/0.21	Echini	Foot layer slightly thickened around apertures [absent ^1^] endexine slightly thicker
*Longetia* (1) *L. buxoides*	Oblate	Spheroid	18–27 (P) x 24–30 (E)	Stephano(6–8)porate	Nanoechinate; psilate in areas between microechini	NO	Thin, continuous-compact/0.14	Absent	Columellate to granular, (short irregular columellae or granula)	Very thick eutectum with only few perforations/0.83	Nanoechini, only few	Infratectum thickened, endexine also slightly thickened
*Mischodon* (4) *M. hexandrum*	Isodiametric	Sphere	34 (D)	Panto(30–50)porate	Echinate, granulate and foveolate to perforate in areas between echini	3–4	Very thin, discontinuous (?)	Thick, continuous/1.5–1.73	Columellate/0.6	Thin, perforated eutectum/0.4	Echini	Foot layer only slightly thicker around aperture
*Mischodon* (1) *M. zeylanicus*	Isodiametric to oblate	Sphere to spheroid	28–34 (P) x 30–38 (E)	Stephano(5–7)porate	Echinate; nanogemmate to granulate in areas between echini	2.5–4.5	Thin continuous-compact/0.17	Absent	Thin, columellate to granular/0.33	Thick, perforated eutectum, palisade-like/0.44–0.76	Echini, baculae	Endexine thickened towards the aperture
*Neoroepera* (2) *N. banksii*	Isodiametric	Sphere	27–36 (D)	Panto(16–40)porate	Echinate; nanobaculate to granulate and perforate in areas between echini	3.7–6	Thin, extending as thin layer across aperture	Thick, continuous/1.0	Columellate, thick columellae/0.7	Thin, perforated semi- to eutectum/0.26–0.3	Echini	Foot layer thickened
*N. buxifolia*							Absent	Thin, discontinuous/0.05	Thin, columellate	Thick, perforated, eu- to semitectum, palisade-like/0.63–0.7	Echini	No ektexinous thickenings
*Oldfieldia* (4) *O. africana*	Isodiametric to oblate	Sphere to spheroid	24–35 (P) x 26–38 (E)	Stephano(5–8)porate	Echinate; nanogemmate to nanorugulate to granulate in areas between echini	2–6	Thin bilayered/0.12, very thin compact outer layer and inner spongy layer	Absent	Columellate/0.69	Perforated eutectum, internal tectum, palisade like/0.38–0.44	Echini	Thickenings absent
*Parodiodendron* (1) *P. marginivillosum*	Oblate	Spheroid	24 (P) x 27 (E)	Stephano(7) porate	Echinate; verrucate to baculate and perforate in areas between echini	3.1	Condition unclear, thin layer^,^ misinterpreted as foot layer ^2^	Thin, continuous/0.1, (absent ^1^)	Columellate/0.4–0.5	Thick, semi- to eutectum, internal tectum, palisade-like/1.1	Echini (baculate in between echini)	Foot layer thickened
*Petalostigma* (5) *P. quadriloculare*	Isodiametric to oblate	Sphere to spheroid	25–32 (P) x 28–34 (E)	Stephano(5–6)porate	Microechinate; nano- to microbaculate and perforate in areas between echini	0.7–1	Absent, [previously misinterpreted ^2^]	Thin, continuous/0.1, (absent ^1^)	Columellate to granular/0.3–0.4	Thick, perforated eutectum, palisade like/4.45–0.51	Microechini	Foot layer thickened around apertures (absent ^1^)
*Picrodendron* (1) *P. baccatum*	Oblate	Spheroid	26 (P) x 29 €	Stephano(5–8)porate	Echinate; microbaculate and perforate in areas between echini	3	Thin, extending as thin layer across aperture	Thin, discontinuous	Columellate (half as thick as tectum)	Thick, perforated/0.6–0.8	Echini	Foot layer slightly thickened towards apertures
*Piranhea* (4) *P. trifoliolata*	Isodiametric to oblate	Sphere to spheroid	21–39 (P) x 24–40 (E)	Stephano(6–8)porate	Echinate; fossulate, perforate, and nanogemmate in areas between echini	1.5–2.9	Very thin continuous/0.04	Discontinuous to absent, [absent ^1^]	Columellate/0.35–0.58	Perforated eutectum, internal tectum, palisade-like/0.47	Echini	NO
*P. mexicana (Synonym: Celaenodendron mexicanum)*							Very thin continuous/0.04, thicker at aperture	Discontinuous to absent [absent ^1^]	Columellate/0.2–0.36	Perforated eutectum, internal tectum (?), palisade-like/0.48–0.72	Echini	Foot layer and endexine thickened at apertures
*Podocalyx* (1) *P. loranthoides*	Isodiametric to oblate	Sphere to spheroid	22–23 (P) x 24–25 (E)	Stephano(3–4)porate	Echinate, perforate in areas between echini	2.5–2.8	Endexine compact, continuous/0.17	Thin, discontinuous/0.17	Very thin, columellate/0.09	Thick, perforated eutectum/0.33	Echini	Endexine thickened towards apertures
*Pseudanthus* (9) *P. divaricatissimus*, *P. ligulatus*, *P. micranthus*, *P. orbicularis*, *P. orientalis*, *P. ovalifolius*, *P. pauciflorus*, *P. pimeleoides*	Isodiametric	Sphere	12.6–27.1 (D)	Panto(5–19)porate	Echinate; foveolate to perforate, psilate to nanoverrucate to granular in areas between echini	0.5–1.8	Thin, discontinuous, extending across the aperture	Thick, continuous/0.54–0.72	Columellate/0.54–0.63	Thin, perforated eutectum/0.09–0.18	Echini	Foot layer thickened around apertures
*Sankowskya* (1)	NO	NO	NO	NO	NO	NO	NO	NO	NO	NO	NO	NO
*Scagea* (2) *S. oligostemon*	Isodiametric	Sphere	30 (D)	Panto(16–20)porate	Echinate; granulate and perforate in areas between echini	1.9	Very thin, continuous/>0.1	Thick, continuous/0.8	Thin, collumelate to granular/0.23	Thin, perforated eutectum/0.15	Echini	Foot layer and endexine slightly thickened at apertures
*Stachyandra* (4) *S. merana*	Isodiametric to oblate	Sphere to spheroid	33–40 (P) x 32–46 (E)	Stephano(4–7)porate [pori often at irregular intervals, 1 or 2 pori can be outside equator]	Microechinate, granulate and perforate in area between echini	0.7–1.4	Absent in interapertural area, present in aperture only	Thin, continuous/0.07 [absent ^1^]	Columellate/0.39	Thick, perforated eutectum/0.39–0.52	Echini	Endexine thickened at apertures
*S. rufibarbis*							Very thin continuous, thicker at aperture	Thin continuous/0.04, only slightly thicker at aperture [absent ^1^]	Thin, columellate/0.16–0.2	Thick, perforated eutectum/0.50–0.56	Microechini	Tectum, foot layer and endexine slightly thicker at aperture
*Stachystemon* (10) *S. polyandrus*	Isodiametric	Sphere	22–28 (D)	Panto(12–22)porate	Echinate; granulate and perforate in areas between echini	1.8	Unclear, not clearly visible (?)	Moderate thick, continuous/0.4	Columellate /0.43	Thin, perforated eutectum/0.1	Echini	Foot layer thickened at apertures
*Tetracoccus* (5) *T. dioicus*	Isodiametric to oblate	Sphere to spheroid	27–46 (P) x 29–47 (E)	Stephano(4–7)porate	Echinate; nanogemmate to granulate, perforate in areas between echini	1.1–5	Very thin, continuous/>0.1	Thin, more or less continuous/0–0.35	Thick, columellate/0.67	Perforated semi- to eutectum, palisade-like /0.8–1.1	Echini (baculae ?)	NO
*Voatamalo* (2) *V. eugenioides*	Isodiametric to oblate	Sphere to spheroid	28–44 (P) x 28–44 (E)	Stephano(5–7)porate	Echinate; nanogemmate to nanorugulate in areas between echini	2.5–4.0	Thin, discontinuous/0.09	Thin, discontinuous/0–0.13	Columellate to granular/0.25–0.48	Thick, perforated semi- to eutectum, palisade-like/0.17–0.65	Echini	Endexine thickened towards apertures
*Whyanbeelia* (1) *W. terrae-reginae*	Oblate	Spheroid	27 (P) x 30 (E)	Stephano(5–6)porate	Echinate; verrucate and perforate in areas between echini	4.1	Unclear (maybe misinterpreted with foot layer ^2^)	Very thin, more or less continuous/0.1 [absent ^2^]	Columellate/0.36	Perforated eutectum/0.45–0.64	Echini	Ektexinous thickenings of basal layer (unclear whether foot layer or endexine)

Note: ^1^ = according to Levin and Simpson [6], ^2^ = according to Simpson and Levin [9]. All measurements are given in µm. Measurements of pollen grain diameters are provided for a single axis (D) or both the polar (P) and equatorial (E) axes. NO = Not observed. Measurements and morphological/ultrastructural traits other than this study are based on illustrations and descriptions compiled from Erdtman [4], Punt [5,17], Köhler [8], Martin [14], Hayden et al. [15], Levin and Simpson [6], Simpson and Levin [9], APSA Members [18], Grímsson et al. [16], and Till et al. [7]. The taxonomy from previous publications has been updated according to POWO [2] and the original LM-, SEM-, and TEM-based terminology has been updated according to Halbritter et al. [13]. The TEM micrographs provided by Levin and Simpson [6] and Simpson and Levin [9] were used to re-measure pollen wall layers and ensure a correct division into foot layer, infratectum, and tectum.

## Data Availability

All data produced for this study are provided in the article.

## References

[1] Centre for Australian National Biodiversity Research APC The Australian Plant Census, IBIS Database. http://www.chah.gov.au/apc/index.html.

[2] POWO Plants of the World Online. Facilitated by the Royal Botanic Gardens, Kew. https://powo.science.kew.org/.

[3] Halford D.A., Henderson Summary Halford R.J., Henderson R.J. (2003). Studies in Euphorbiaceae A.L.Juss. *sens. lat.* 5. A revision of *Pseudanthus* Sieber ex Spreng. and *Stachystemon* Planch. (Oldfieldioideae Köhler & Webster, Caletieae Müll.Arg.). Austrobaileya.

[4] Erdtman G. (1952). Pollen Morphology and Plant Taxonomy.

[5] Punt W. (1962). Pollen Morphology of the Euphorbiaceae with Special Reference to Taxonomy. Wentia.

[6] Levin G.A., Simpson M.G. (1994). Phylogenetic Implications of Pollen Ultrastructure in the Oldfieldioideae (Euphorbiaceae). Ann. Mo. Bot. Gard..

[7] Till A., Ulrich S., Cantrill D.J., Grímsson F. (2026). Pollen Morphology of *Pseudanthus* (Picrodendraceae). Rev. Palaeobot. Palynol..

[8] Köhler E. (1965). Die Pollenmorphologie Der Biovulaten Euphorbiaceae Und Ihre Bedeutung Für Die Taxonomie. Grana.

[9] Simpson M.G., Levin G.A. (1994). Pollen Ultrastructure of the Biovulate Euphorbiaceae. Int. J. Plant Sci..

[10] Ulrich S., Purgina C., Bouchal J.M., Geier C., Grímsson F. (2026). The Use of Transmission Electron Microscopy When Investigating Fossil Angiosperm Pollen: A Review and Suggestions for Future Applications. Rev. Palaeobot. Palynol..

[11] The Angiosperm Phylogeny Group (2016). An Update of the Angiosperm Phylogeny Group Classification for the Orders and Families of Flowering Plants: APG IV. Bot. J. Linnéan Soc..

[12] Sutter D.M., Forster P.I., Endress P.K. (2006). Female Flowers and Systematic Position of Picrodendraceae (Euphorbiaceae s.l., Malpighiales). Plant Syst. Evol..

[13] Halbritter H., Ulrich S., Grímsson F., Weber M., Zetter R., Hesse M., Buchner R., Svojtka M., Frosch-Radivo A. (2018). Illustrated Pollen Terminology.

[14] Martin H.A. (1974). The Identification of Some Tertiary Pollen Belonging to the Family Euphorbiaceae. Aust. J. Bot..

[15] Hayden W.J., Gillis W.T., Stone D.E., Broome C.R., Webster G.L. (1984). Systematics and Palynology of Picrodendron: Further Evidence for Relationship with the Oldfieldioideae (Euphorbiaceae). J. Arnold Arbor..

[16] Grímsson F., Graham S.A., Coiro M., Jacobs B.F., Xafis A., Neumann F.H., Scott L., Sakala J., Currano E.D., Zetter R. (2019). Origin and Divergence of Afro-Indian Picrodendraceae: Linking Pollen Morphology, Dispersal Modes, Fossil Records, Molecular Dating and Paleogeography. Grana.

[17] Punt W. (1987). A Survey of Pollen Morphology in Euphorbiaceae with Special Reference to *Phyllanthus*. Bot. J. Linn. Soc..

[18] APSA Members* 2007 Australasian Pollen and Spore Atlas. https://apsa.anu.edu.au/.

[19] Hayden W.J. (1994). Systematic Anatomy of Euphorbiaceae Subfamily Oldfieldioideae I. Overview. Ann. Mo. Bot. Gard..

[20] Ulrich S., Grímsson F. (2020). The Single-Grain Method: Adding TEM to the Equation. Grana.

[21] Weber M., Ulrich S. (2010). The Endexine: A Frequently Overlooked Pollen Wall Layer and a Simple Method for Detection. Grana.

